# Efficacy of Physiotherapy Rehabilitation for Proximal Femur Fracture

**DOI:** 10.7759/cureus.30711

**Published:** 2022-10-26

**Authors:** Nidhi Tiwari, Shubhangi Patil, Rupali Popalbhat

**Affiliations:** 1 Physical Therapy, Ravi Nair Physiotherapy College, Datta Meghe Institute of Medical Sciences, Wardha, IND; 2 Community Physiotherapy, Ravi Nair Physiotherapy College, Datta Meghe Institute of Medical Sciences, Wardha, IND

**Keywords:** physiotherapy intervention, physiotherapy, proximal femur fracture, fracture, rehabilitation

## Abstract

A diaphysis fracture that occurs between 5 cm distal to the lesser trochanter and 5 cm proximal to the adductor tubercle is recognized as a femoral shaft fracture and is prevalent in runners and military personnel. A patient's ability to carry out activities of daily living effectively and efficiently post-surgery is hampered by a variety of obstacles. We present a case of a 21-year-old male who came to the hospital with a complaint of pain and swelling in his right leg. The patient was diagnosed with a proximal femur fracture. Physiotherapy procedures commenced with the purpose of alleviating pain and establishing a normal range of motion. As a result of the physiotherapy regimens, the patient was aided in his recovery.

## Introduction

Proximal femoral fractures involve femoral neck fractures which are a subgroup of proximal femoral fractures. The weakest region of the femur is the femoral neck [1]. In light of the large population, high prevalence of traffic accidents, and rising average age of the population, the proximal femoral fracture is a major cause of morbidity and mortality worldwide [2]. Different treatment approaches are needed for many adult proximal femoral fracture types, depending on factors such as the fracture's location, morphologic characteristics, mechanism of injury, stability, patient age, and baseline functional level [3]. These fractures frequently originate from high-energy causes such as motor vehicle crashes (MVC), which can lead to malformations and limb shortening. Femoral shaft fractures (FSF) often have a bimodal distribution, with younger people typically experiencing high-energy trauma and older people experiencing lower-energy trauma [4]. Demographic traits, injury severity and mechanism, and fracture site all have an impact on the pattern, presentation, and treatment of femoral fractures [5]. A significant portion of trauma-related hospitalizations is due to proximal femoral fractures. The vast majority of these patients are over the age of 50 (>90%). These fractures affect females two to three times more than males. The neck of femur fractures, intertrochanteric fractures, and subtrochanteric fractures are the three types based on the anatomical position of the fracture. Each of these fracture types necessitates unique treatment procedures [6].

## Case presentation

**Figure 1:**
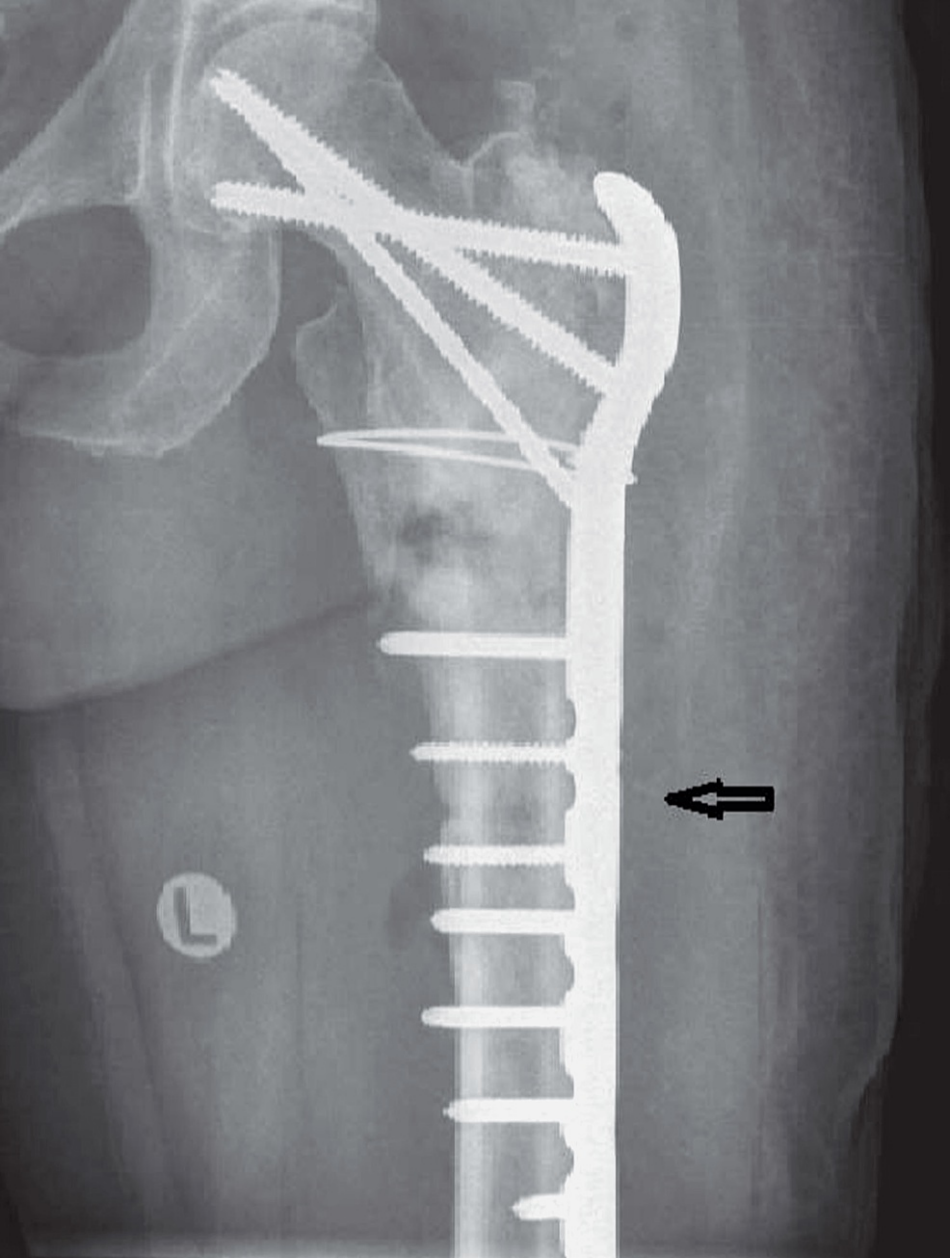
The fracture was treated with open reduction and internal fixation (ORIF). Plates were used as internal fixator to treat the fractured femur.

A 21-year-old male had a history of road traffic accident (RTA) due to which he had a femur fracture. He was brought to the emergency department of a rural hospital for an X-ray. Swelling and grade 2 tenderness were present in his leg. His range of motion (ROM) and manual muscle testing (MMT) was decreased. The proximal femur fracture was corrected with an open reduction and internal fixation approach shown in Figure 1. He was placed in the supine position on the bed, as it was the most comfortable position.

Physiotherapy intervention

To reinstate hip and knee movements to normal, or at the very least to a functional ROM to improve and regain the strength of hip movements, and to restore ROM for hip and knee joints, the patient underwent physiotherapy (Table 1).

**Table 1:** Therapeutic intervention ROM: Range of motion, CPM: Continuous passive movement, ADL: Activity of daily living

Week 1	Weeks 2 to 4	Weeks 4 to 8	After 8 weeks
Full active ROM to the ankle with gentle active movement of hip and knee to the left side. Isometrics glutei and quadriceps exercises. Ankle-toe movement.	Active and active assisted movements for the hip, knee, and ankle. Ankle isotonic and isometric to hip and knee.	Hip flexion up to 90 degrees, and self-assisted heel slides. Bedside sitting with the leg hanging with unaffected extremity supporting the affected one. Assisted and self-resistive exercise for the hip and knee.	Passive ROM by therapist or CPM machine. Isotonic with Isokinetic exercises for the hip and knee along with resistive exercise. The ADL is done with assistive devices.
Non-weight bearing is initiated using a walker with a three-point gait.	Non-weight bearing using a walker with a three-point gait.	Gradual weight bearing. From prone lying to four-point kneeling. The patient bears weight on the knee and then knee walking is encouraged. Partial weight bearing by using a walker with a three-point gait is initiated.	Full weight bearing is initiated along with a walker with a four-point gait.

Follow-up and outcome measure

After proper rehabilitation, the patient's ROM i.e., both active and passive, was increased at the time of discharge (Table 2). An increase in muscle strength was also observed via pre- and post-manual muscle testing (MMT) (Table 3) [7]. 

**Table 2:** Active and passive range of motion before and at the time of discharge. AROM: Active range of motion PROM: Passive range of motion

Joint	AROM		PROM	
Hip	Baseline	Discharge	Baseline	Discharge
Flexion	80	90	83	95
Extension	10	20	17	27
Adduction	10	19	12	20
Abduction	15	25	18	28
Knee				
Flexion	30	65	34	69

**Table 3:** Pre- and post-treatment MMT MMT: Manual muscle testing

Pre-treatment	Post-treatment
1	+3

## Discussion

In the elderly, femur fractures are the leading cause of morbidity, hospitalizations, and mortality after hip fractures. Furthermore, a large percentage of these patients do not regain their pre-fracture functional status. Only about half of the survivors can walk without assistance a year after surgery, and only around 40% can do independent activities of daily living (ADL). Knowing this, the goal of physical therapy in the postoperative treatment of proximal femoral fracture patients is to enhance muscular strength and the ability to walk safely. As a consequence, geriatric patients can become more efficient and independent.

Carneiro et al. in their study stated that physical therapy after fracture repair is crucial because it emphasizes early mobilization, gait training, and other treatment modalities to maintain or restore potential impairments. Physiotherapy intervention has a positive impact to gain more confidence [8]. Paterno et al. in a study stated that femoral fractures that have had intramedullary nailing consistently heal. However, functional limits and impairments frequently last more than a year after surgery, making it difficult for the patient to resume normal daily activities, gait, or job. To reduce handicaps, medical and therapeutic therapy should encourage a quick and secure return to function [9]. Lacerations, abrasions, and avulsions are examples of soft tissue injuries. And physiotherapy helps patients to improve their functional status and gain confidence.

## Conclusions

Our patient's ROM and muscle strength in the lower limb and face muscles were enhanced with physiotherapy. By the end of the session, the prognosis had eased. Both the patient and doctor were pleased with the outcome at the time of discharge. The patient would have been better served if he or his family had been enlightened about the need for physiotherapy before any surgery.
